# G-YOLO: A YOLOv7-based target detection algorithm for lightweight hazardous chemical vehicles

**DOI:** 10.1371/journal.pone.0299959

**Published:** 2024-04-24

**Authors:** Cuiying Yu, Lei Zhou, Bushi Liu, Yue Zhao, Pengcheng Zhu, Liqing Chen, Bolun Chen

**Affiliations:** 1 Faculty of Computer and Software Engineering, Huaiyin Institute of Technology, Huaian, China; 2 Fujian Provincial Key Laboratory of Network Security and Cryptology, Fuzhou, China; 3 Department of Physics, University of Fribourg, Fribourg, Switzerland; VIT-AP Campus, INDIA

## Abstract

Hazardous chemical vehicles are specialized vehicles used for transporting flammable gases, medical waste, and liquid chemicals, among other dangerous chemical substances. During their transportation, there are risks of fire, explosion, and leakage of hazardous materials, posing serious threats to human safety and the environment. To mitigate these possible hazards and decrease their probability, this study proposes a lightweight object detection method for hazardous chemical vehicles based on the YOLOv7-tiny model.The method first introduces a lightweight feature extraction structure, E-GhostV2 network, into the trunk and neck of the model to achieve effective feature extraction while reducing the burden of the model. Additionally, the PConv is used in the model’s backbone to effectively reduce redundant computations and memory access, thereby enhancing efficiency and feature extraction capabilities. Furthermore, to address the problem of performance degradation caused by overemphasizing high-quality samples, the model adopts the WIoU loss function, which balances the training effect of high-quality and low-quality samples, enhancing the model’s robustness and generalization performance. Experimental results demonstrate that the improved model achieves satisfactory detection accuracy while reducing the number of model parameters, providing robust support for theoretical research and practical applications in the field of hazardous chemical vehicle object detection.

## Introduction

With the rapid economic development in China, there has been a continuous increase in the circulation of hazardous materials across various sectors, particularly in key areas such as energy, raw materials, and consumer goods. According to the latest statistical data, the daily transportation volume of hazardous chemicals in China has exceeded 1 million tons, with an annual total transportation volume surpassing 400 million tons [1]. This highlights the integral role of hazardous chemical transportation in China’s economic system [2]. The widespread production and use of hazardous chemicals have added complexity to their transportation activities, with hazardous chemical vehicles playing a crucial role in transporting these substances from production sites to end users, involving extensive logistics networks and transportation systems. However, as the number of hazardous chemical vehicles on the roads continues to rise, there is an increasingly urgent demand for their safety management and monitoring.

During the transportation of hazardous chemical vehicles, there are inherent risks, including fire, explosions, and the leakage of hazardous substances [3]. These risks not only pose a direct threat to human health but also have the potential to cause harm to the environment. Therefore, real-time monitoring of hazardous chemical vehicles becomes a crucial means to ensure traffic safety and safeguard the lives and properties of the people. Real-time monitoring not only aids in preventing accidents but also enables the early detection of potential issues, allowing for timely measures to be taken to minimize the impact of accidents on the environment and individuals. In this context, there is an urgent need for an efficient and reliable monitoring system to ensure the safe transportation of hazardous chemical vehicles, thereby maintaining societal stability and promoting sustainable development.

With the rapid advancement of artificial intelligence technology, target detection algorithms based on deep learning have made remarkable progress in the field of hazardous chemical vehicle detection [4]. However, this field still faces a series of significant challenges, with real-time performance and accuracy being the most prominent issues. Hazardous Chemical vehicles typically navigate through complex and dynamic road environments, susceptible to various interference factors such as other vehicles, pedestrians, and road signs. These factors not only complicate target detection but also impose high demands on the real-time capabilities of detection algorithms. In scenarios with high traffic density and complex environments, ensuring that detection algorithms can timely and accurately identify hazardous chemical vehicles is an urgent requirement for ensuring road transport safety. Therefore, addressing the challenges of real-time performance and accuracy becomes a crucial factor in driving forward the technology for detecting hazardous chemical vehicles. Only through in-depth research and innovative algorithm development, overcoming these challenges, can we establish an efficient and reliable monitoring system for hazardous chemical vehicles in real-world road scenarios, contributing to traffic safety and public security. In this process, close integration of advanced deep learning technologies with practical requirements is essential to meet the complexity and high demands of hazardous chemical vehicle detection.

To address the challenges of insufficient real-time performance and accuracy in hazardous chemical vehicle detection, the single-stage YOLO algorithm proves to be a more ideal target detection method. Among them, YOLOv7-Tiny stands out as a lightweight version of the YOLOv7 target detection algorithm [5], possessing advantages in speed and efficiency. However, concerning the task of hazardous chemical vehicle detection, YOLOv7-Tiny has some limitations, such as lower detection accuracy and difficulty in deployment on end devices. Therefore, we have undertaken a series of improvements to YOLOv7-Tiny to overcome the existing limitations. The specific contributions of this paper are as follows:

1. Firstly, by introducing a lightweight feature extraction structure, the E-GhostV2 network, aims to reduce the burden on the model while achieving effective feature extraction. This improved lightweight provides strong support for the deployment of the model on end devices, which in turn enhances its applicability in real-world scenarios. This innovation not only helps reduce the computational requirements of the model during deployment but also provides a viable solution for real-time detection in complex environments and on-end devices.
2. Secondly, by introducing partial convolutions to enhance the original model, it becomes possible to effectively reduce computational redundancy and the number of memory accesses. The introduction of partial convolutions enables the model to more precisely capture critical features, thereby improving detection accuracy in complex scenarios. This innovative design not only makes the model more efficient but also strengthens its robustness in hazardous chemical vehicle detection tasks.
3. Finally, the WIoU loss function is introduced to balance the training effect of high-quality samples and low-quality samples, which effectively solves the problem that the regression frames cannot be accurately matched due to sample quality imbalance during the detection of hazardous chemical vehicles. This strategy of balancing high and low-quality samples helps to make the model more robust and better adapted to the challenges in real environments, which further ensures that the model can perform the task of detecting dangerous chemical vehicles robustly and efficiently in real applications.

## Related work

### Traditional detection methods

Traditional vehicle detection methods include various approaches such as image processing-based methods [6], feature extraction, classifier-based methods, template matching-based methods, and machine learning-based methods.

For instance, Tao et al. proposed a car detection method for low-resolution ship images that utilized body boundaries, front windshield boundaries, and shadows as key features. Although it worked well for vertical and slightly tilted viewpoints, its detection capability was limited for large degrees of tilt, leading to constraints in handling varying viewing angles [7]. Bougharriou et al. introduced a HOG vehicle detection method using linear SVM classifiers with oriented gradient feature descriptors and histograms. While this method showed promising results in vehicle detection, it suffered from low system performance, lacking real-time capability and robustness [8]. Wei et al. proposed a new image strip feature for vehicle detection, employing an integral image-based feature extraction method and a complexity-aware criterion of the RealBoost framework. This method improved efficiency while maintaining accuracy. However, compared to complex descriptors like HOG and covariance descriptors, the image strip feature discarded some statistical information, affecting its discriminative ability [9].

In summary, traditional vehicle detection methods have limitations, including sensitivity to changes in viewing angle, challenges in handling complex backgrounds and occluded targets, low real-time performance, and reduced accuracy in recognition.

### Deep learning-based detection methods

Deep learning-based target detection models [10] can be categorized into two main types: two-stage target detection and one-stage target detection. Two-stage detection algorithms, such as R-CNN [11], Fast R-CNN [12], Faster R-CNN [13], and Mask RCNN [14], first extract candidate regions and then perform classification and bounding box regression. These methods achieve high accuracy but are relatively slow in processing speed. On the other hand, one-stage detection algorithms, like the YOLO (You Only Look Once) family [15] and SSD [16], predict target location and class directly through a single network, providing real-time and fast detection capabilities.

Kuang et al. developed a deep learning-based highway vehicle detection method that utilizes a single-shot multi-box target detector (SSD) to mark vehicles in highway videos. They trained the vehicle detector using SSD and tested the onboard detector for vehicle detection [17]. Manana et al. introduced a preprocessed Faster R-CNN for road vehicle detection, incorporating Sobel edge operators and Hough transform-based preprocessing lane pipelines for lane detection. This improved the training and detection speed of the model [18]. Piedad et al. introduced a vehicle counting system based on deep learning with Mask R-CNN. They compared its output to the conventional approach of manually recording past vehicle data, demonstrating higher accuracy in classifying and quantifying automobile-type vehicles using the developed tool [19]. Bie et al. proposed an improved lightweight vehicle detection algorithm based on YOLOv5. The algorithm combines depth separable convolutions and the C3Ghost module, replacing the original C3 module, aiming to reduce model parameters and enhance detection speed [20]. Murthy et al. introduced LD-CNNs, a lightweight deep convolutional neural network detection model. The detection algorithm incorporates a multi-condition constraint generative adversarial network named MC-GAN, which efficiently generates samples, significantly reducing the model’s computational cost and improving detection accuracy [21]. Talaat, F.M. and ZainEldin, H. introduced a novel approach for fire detection in smart cities utilizing the YOLOv8 Smart Fire Detection System (SFDS). SFDS leverages deep learning to detect fire-specific features in real-time, aiming to enhance accuracy and reduce false alarms compared to traditional methods. The proposed framework integrates Fog and Cloud computing, along with the IoT layer, for real-time data collection and processing, ensuring faster response times and mitigating risks. SFDS demonstrates state-of-the-art performance with a high precision rate of 97.1% for all classes. The proposed approach holds potential applications in fire safety management, forest fire monitoring, and intelligent security systems [22]. Chen et al. designed a novel convolutional algorithm using group convolutions and introduced new criteria to determine the effective range of groups. Based on these new design criteria, they developed a lightweight detection network called DenseLightNet. The detector designed using this network achieved a threefold increase in operational speed compared to YoloV3, with a smaller model size [23]. Li et al. proposed a 3D object detector named 6DoF-3D, which adopts an efficient and accurate single-stage LiDAR-based detection approach. The network structure includes the CSPDarknet53 backbone, multi-scale feature fusion neck, and detection head. By converting 3D point clouds into pseudo-images and employing a novel encoding strategy, this model successfully reduces the computational cost of the network [24].

Although the above deep learning-based vehicle detection methods have achieved good results, they are still prone to issues like missed detections and false detections in the presence of target occlusion and complex scenes. Additionally, large models are unsuitable for mobile deployment and fail to meet real-time demands.

## Description of the problem

Detecting hazardous material vehicles is of significant importance in enhancing safety and emergency response. However, it faces challenges such as complex backgrounds, small-sized targets, diverse categories, and real-time requirements. Overcoming these limitations requires continuous improvement of algorithms and technologies to achieve more accurate and efficient detection of hazardous material vehicles. Therefore, this paper aims to propose a lightweight and efficient object detection algorithm to address these challenges and further enhance the level of hazardous material vehicle detection. The effectiveness of the algorithm is experimentally validated on three different datasets, using various evaluation metrics to determine the model’s performance under different scenarios.

### Datastes

**Fig 1:**
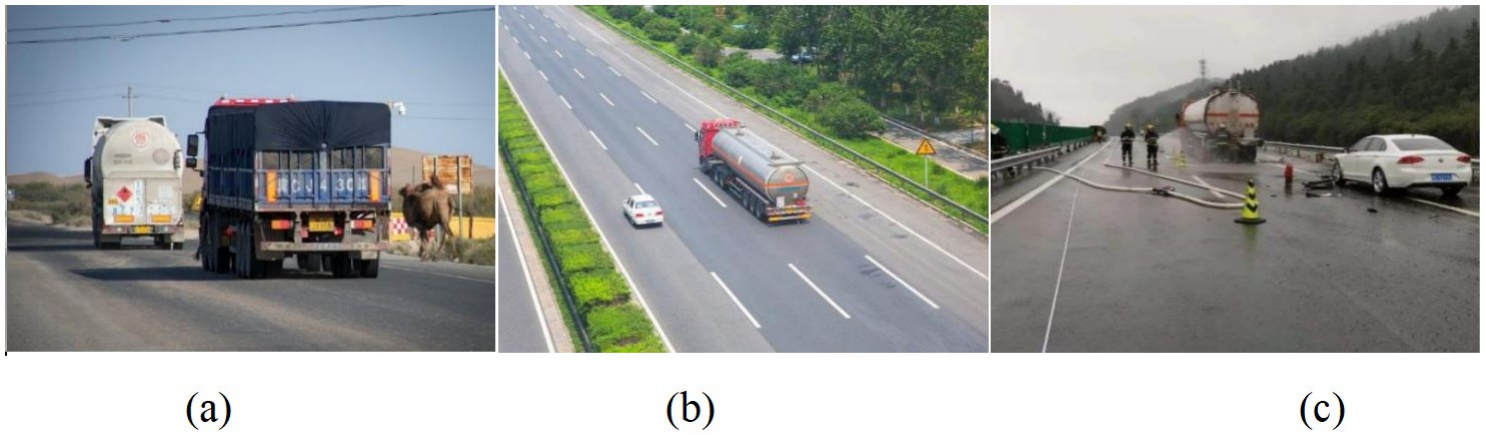
Display of selected datasets. (a)(b)(c)shows a selection of images from the Dangerous Materials Vehicles dataset. Reprinted from [] under a CC BY license, with permission from [Pengcheng Zhu], original copyright [2023].

The experimental dataset used in this paper is the hazardous chemical vehicle dataset [25](open access link https://www.kaggle.com/datasets/yucuiyingcuiyingyu/dataset-for-plos-one), encompassing four categories of vehicle targets: large trucks, oil trucks, manned buses, and tiny cars. The dataset comprises 4363 images, which were divided into training, testing, and validation sets with a ratio of 6:2:2. Specifically, 2617 images were allocated for model training, 873 images for model testing, and another 873 images for model validation. A partial image of the Hazardous Materials Vehicle dataset is shown in Fig 1.

The Cars Detection dataset is a comprehensive dataset showcasing various vehicles, including five different categories: “Ambulance,” “Bus,” “Car,” “Motorcycle,” and “Truck.” This dataset not only includes small-sized motorcycles but also features large-sized trucks, allowing detection models to face different real-world challenges. Additionally, the dataset captures vehicles under various environmental conditions, lighting scenarios, and viewpoints, reflecting the complexity of object detection tasks in practical applications(open access link ). The Traffic Detection dataset includes vehicle images captured from over 100 cities and rural areas, totaling over 11,000 images. It comprises six categories: “Bike”, “Auto”, “Car”, “Truck”, “Bus”, and “Other Vehicles” (Rickshaw, Van, Cycle, etc.). This dataset covers diverse lighting conditions, such as day and night, various weather conditions, different distances, and viewpoints, providing a comprehensive test of the model’s detection performance under complex conditions(open access link ).

### Evaluation indicators

In this paper, several modeling evaluation metrics are employed, including Precision (*P*), Recall (*R*), Intersection over Union (*IoU*), and Mean Average Precision (*mAP*). To clarify the definitions of True Positive (*TP*), True Negative (*TN*), False Positive (*FP*), and False Negative (FN), *TP* is the number of positive samples correctly identified as positive, *TN* is the number of negative samples correctly identified as negative, *FP* is the number of negative samples incorrectly identified as positive, and *FN* is the number of positive samples incorrectly identified as negative.

Precision (*P*) refers to the proportion of correctly identified positive samples among all predicted positive samples. This study specifically denotes the proportion of correctly identified hazardous chemical vehicles among all predicted targets recognized as hazardous chemical vehicles. A higher precision indicates better detection performance of the model. The calculation formula is as follows:

$$\begin{array}{c} P=\frac{TP}{TP+FP} \end{array}$$ (1)

Recall (*R*) refers to the proportion of correctly identified positive samples among all actual positive samples. In this study, it specifically denotes the proportion of actual hazardous chemical vehicle targets that are correctly classified as hazardous chemical vehicles. In object detection tasks, recall evaluates whether the model can comprehensively capture all targets in the image without missing any actual targets. A high recall indicates that the model can effectively find most targets, reducing the likelihood of false negatives (missed detections). The calculation formula is as follows:

$$\begin{array}{c} R=\frac{TP}{TP+FN} \end{array}$$ (2)

Intersection over Union (*IoU*) refers to the ratio of the intersection area to the union area between the *Bbox*(predicted bounding box) and the *Tbox* (ground truth bounding box). It provides a quantitative measure to assess the prediction accuracy of the model. A higher IoU indicates more accurate predictions by the model. The calculation formula is as follows:

$$\begin{array}{c} IoU=\frac{Bbox\cap Tbox}{Bbox\cup Tbox} \end{array}$$ (3)

Mean average precision (*mAP*) is calculated by combining *IoU*, precision, recall, and the confusion matrix, making it one of the most crucial metrics in object detection [26]. It represents the average precision across multiple classes, with values ranging from [0, 1], where higher values indicate better performance. *mAP*@0.5 calculates the Average Precision for each class using *IoU* set to 0.5 and then averages across all classes. *mAP*@.5: .95 represents the average over different thresholds (from 0.5 to 0.95 with a step size of 0.05). The formula for calculation is as follows:

$$\begin{array}{c} mAP=\frac{1}{classnumber}\sum_{1}^{classnumber}AP \end{array}$$ (4)

## Algorithm design

**Fig 2:**
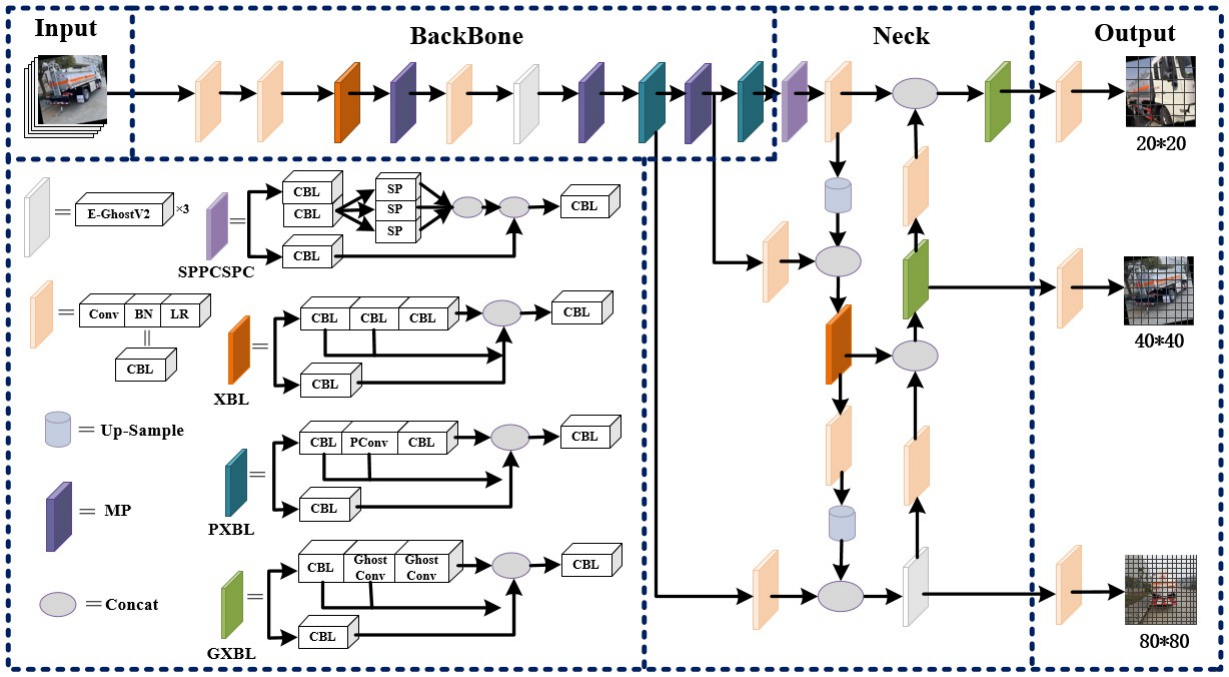
G-YOLO modeling framework.

This paper proposes an improved hazardous chemical vehicle detection algorithm model, G-YOLO, whose model framework is illustrated in Fig 2. The design of this model aims to overcome challenges faced by traditional object detection models in hazardous chemical vehicle detection tasks, with a focus on improving detection accuracy and real-time performance.

The proposed model enhances the detection accuracy of hazardous chemical vehicles in complex and dense scenarios by integrating the lightweight E-GhostV2 network into the backbone and neck. This integration ensures computational efficiency while enabling deployment on end devices and improving real-time performance. Moreover, the utilization of PConv in the backbone significantly reduces computational redundancy and memory accesses, leading to faster and more accurate detection of hazardous chemical vehicles, while minimizing the risk of model leakage and misdetection. Additionally, the WIoU loss function is applied to balance the impact of high-quality and low-quality samples during training, enabling the model to rely more on anchor frame features for precise prediction, adapt better to diverse shapes and positions of hazardous chemical vehicle targets, and improve detection accuracy, particularly for small-sized and occluded targets.

### Lightweight feature extraction network E-GhostV2

Traditional models for hazardous chemical vehicle detection are typically complex and large, requiring significant computational resources and memory, making them challenging to deploy effectively on mobile devices. Simultaneously, capturing the spatial relationships between pixels of hazardous chemical vehicles at long distances in complex backgrounds is a challenging task. In complex backgrounds, there may be numerous interfering objects and occlusions, increasing the difficulty of detecting and locating hazardous chemicals and vehicles.

The Ghost module proposed by GhostNet is a lightweight and efficient convolutional module [27]. It generates more diverse feature maps by utilizing inexpensive linear operations, effectively reducing the parameters and computational load of convolutional layers without changing the output feature map size and channel size. However, the convolution operation can only capture local information in the window region, limiting the improvement in model performance. Introducing the Decoupled Fully Connected Attention (DFC attention) mechanism in convolution can overcome this limitation. It enhances the long-range dependency of feature maps by performing fully connected operations horizontally and vertically on the feature maps, thereby improving the detection performance of the model [28]. The GhostNetV2 network is constructed by stacking Ghost modules and the Decoupled Fully Connected Attention mechanism. Its design achieves a good balance between lightweight and high performance, providing strong support for efficient deployment of the model in resource-constrained environments.

**Fig 3:**
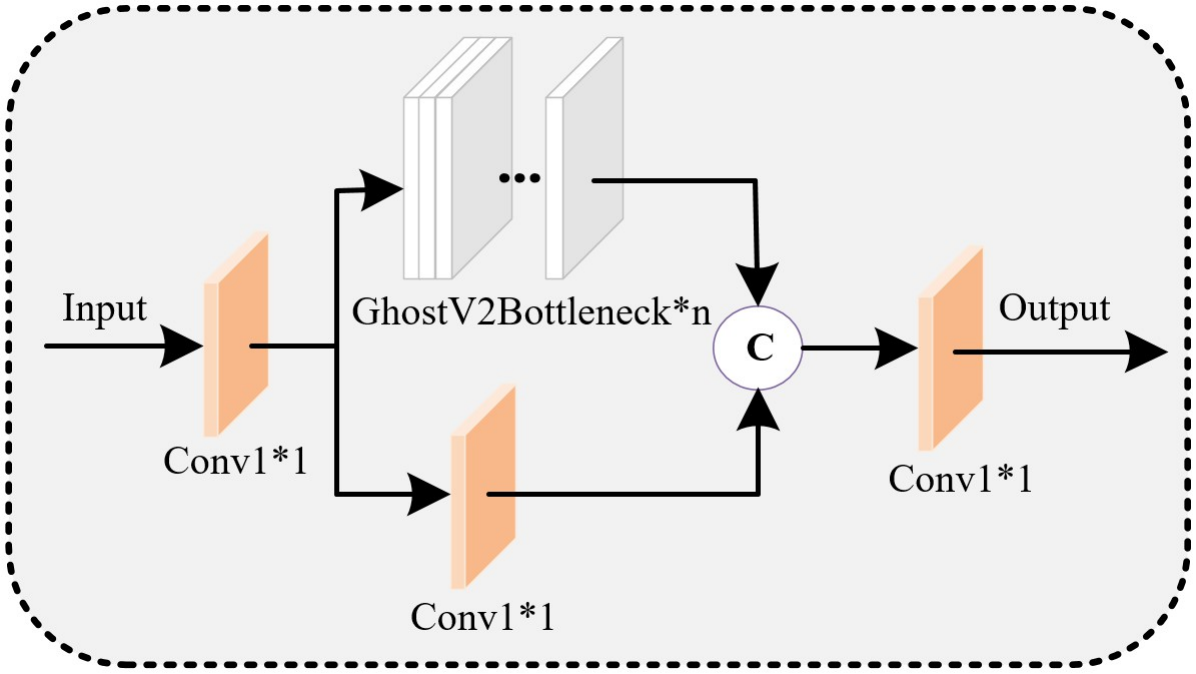
E-GhostV2 network diagram.

Leveraging the structural characteristics of GhostNetV2, we designed a lightweight feature extraction network called E-GhostV2. Introducing the E-GhostV2 network into the YOLOv7-Tiny object detection network model allows for effective feature extraction while reducing the model’s burden. The schematic diagram of the E-GhostV2 network is illustrated in Fig 3.

**Fig 4:**
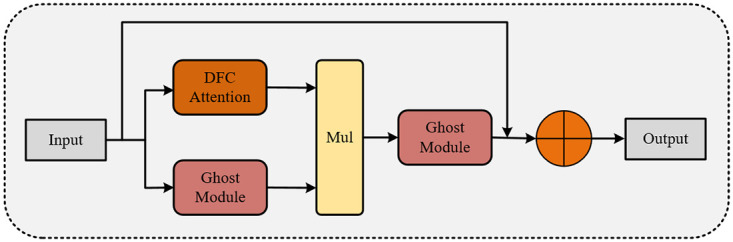
GhostV2Bottleneck.

The GhostV2Bottleneck consists of two Ghost modules and one DFC attention mechanism, as depicted in Fig 4. The first Ghost module aims to expand the number of feature channels, while the second Ghost module generates the output features by reducing the channel count. The advantage of this design is the effective decoupling of the model’s expressive power and computational complexity, addressing potential overfitting issues during training and insufficient generalization during testing. The DFC attention mechanism is introduced to capture long-range correlations between different spatial position pixels. The DFC attention mechanism operates in parallel with the first Ghost module to enhance features, and the second Ghost module receives the enhanced features to generate output features, further boosting model performance. This structural design contributes to maintaining model performance while enhancing its generalization ability. Below is a detailed introduction to the Ghost module and DFC attention mechanism and their functionalities.

**Fig 5:**
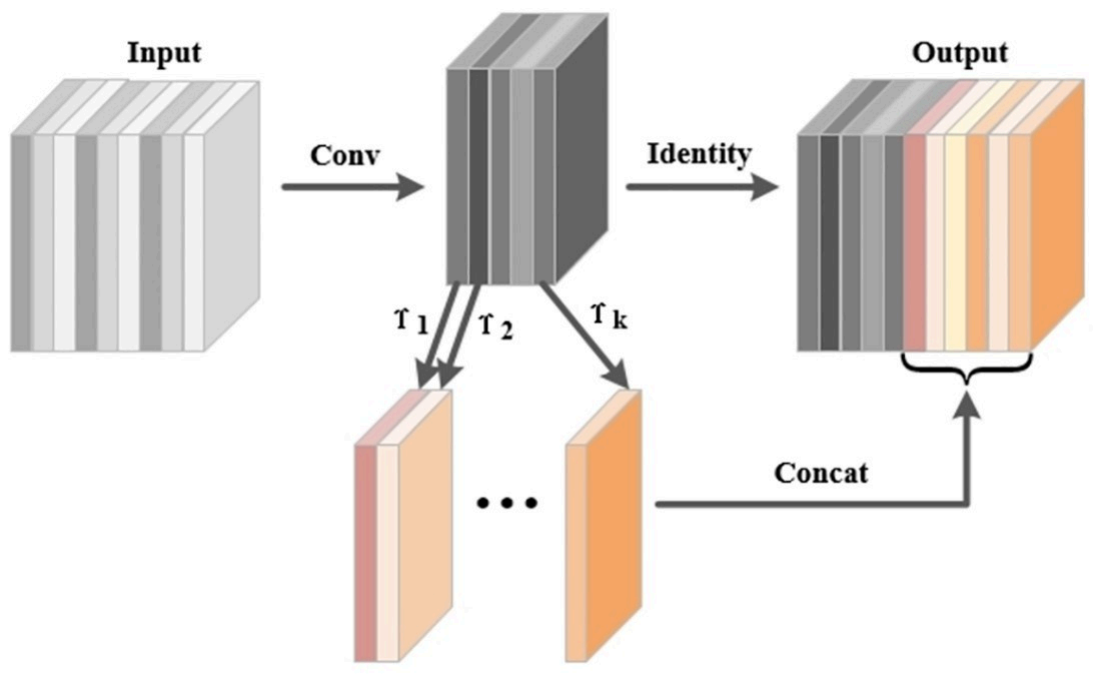
Ghost module.

The Ghost module is a lightweight convolutional neural module that effectively generates more feature maps with fewer parameters(as shown in Fig 5). Given the input features *X* = *R*^*H* × *W* × *C*^(*H*, *W*, and *C* denote the height, width, and number of channels of the feature map, respectively), the Ghost module divides the output channel into two parts. First, the input features *X* undergo a 1 × 1 pointwise convolution to obtain a portion of the output features*Y*′. Subsequently, this is used as input to generate another set of features through a 3 × 3 depthwise convolution. Finally, the outputs from these two parts are concatenated to obtain the ultimate output, as computed in Eqs (5) and (6):

$$\begin{array}{c} Y\prime =X*F_{11} \end{array}$$ (5)

$$\begin{array}{c} Y=Concat(Y\prime ,Y\prime *F_{d}) \end{array}$$ (6)

In the equations mentioned above,$Y\prime \in R^{H\times W\times C_{out}^{\prime }}$, and * denotes the convolution operation. *F*_11_ denotes the 1*1 pointwise convolution, *Concat* denotes the splicing operation, *F*_*d*_ represents the 3 × 3 depthwise convolution, and $Y\in R^{H\times W\times C_{out}}$ denotes the final output features. The Ghost module, compared to regular convolution modules with the same input and output feature map quantities, successfully achieves a significant reduction in parameters and computations. However, this inevitably leads to a decrease in its feature representation capability. In the Ghost module, only a portion of features is input into the 3 × 3 depthwise convolution to capture spatial features, while the rest of the features undergo a 1 × 1-pointwise convolution operation to reduce computational complexity. The relationship between spatial pixels is crucial for accurate recognition. However, pointwise convolution operations do not handle the spatial features of the input tensor; they only perform convolution operations on channels. Therefore, this limits the module’s ability to capture spatial information, hindering further performance improvement.

The DFC attention module is employed to enhance the output features of the Ghost module, thereby strengthening the model’s ability to capture distant information between different spatial pixels. Specifically, the DFC attention mechanism designs a fully connected layer with fixed weights to generate an attention map with a global receptive field. Firstly, the given input *Z* ∈ *R*^*H* × *W* × *C*^ is considered as *H* × *W* tokens, denoted as *Z* ∈ {*Z*_11_, *Z*_12_, …, *Z*_*HW*_}. Next, it is decomposed into two fully connected layers along the horizontal and vertical directions, respectively. This decomposition allows the model to effectively perceive long-distance dependencies, as represented in the following formulas:

$$\begin{array}{c} \alpha_{hv}\prime =\sum_{h\prime =1}^{H}F_{h,h\prime w}^{H}*Z_{h\prime w},h=1,2,...,H,w=1,2,...,W \end{array}$$ (7)

$$\begin{array}{c} \alpha_{hv}=\sum_{h\prime =1}^{W}F_{h,h\prime w}^{W}*\alpha_{hv}\prime ,h=1,2,...,H,w=1,2,...,W \end{array}$$ (8)

**Fig 6:**
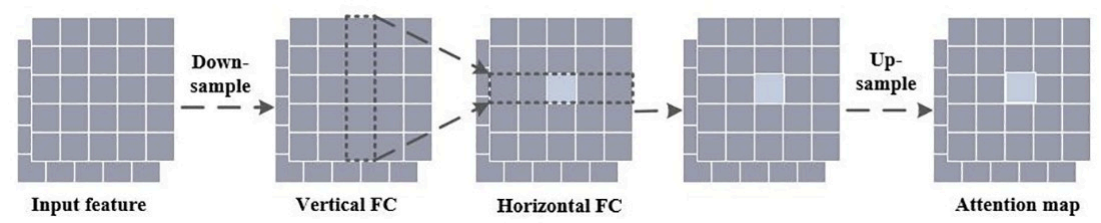
Decoupling fully connected attention.

Where *F*^*W*^ and *F*^*H*^ represent the learning weights. The output *α*_*hv*_′ represents the attention along the vertical direction, while *α*_*hv*_ represents the fusion of the attention along both horizontal and vertical directions, yielding the complete attention output. After applying Eqs (7) and (8), the DFC attention mechanism is obtained, as illustrated in Fig 6.

### Partial convolution

In traditional target detection tasks for hazardous chemical vehicles, the overheads of computation and memory access tend to have an impact on the model accuracy. If the latency exceeds the requirements of a specific application scenario, the problem of wrong detection and missed detection may occur. Meanwhile, in dealing with large-scale dense vehicles or real-time scenarios, if the model is unable to meet the required high throughput requirements, it will lead to a reduction in the efficiency of the model in the training and inference phases, which will affect the model’s accuracy for hazardous chemical vehicles. To solve this problem, we use PConv to improve the network so that the hazardous chemical vehicle detection model has higher efficiency and better feature extraction capability, thus improving the accuracy and precision of detection.

**Fig 7:**
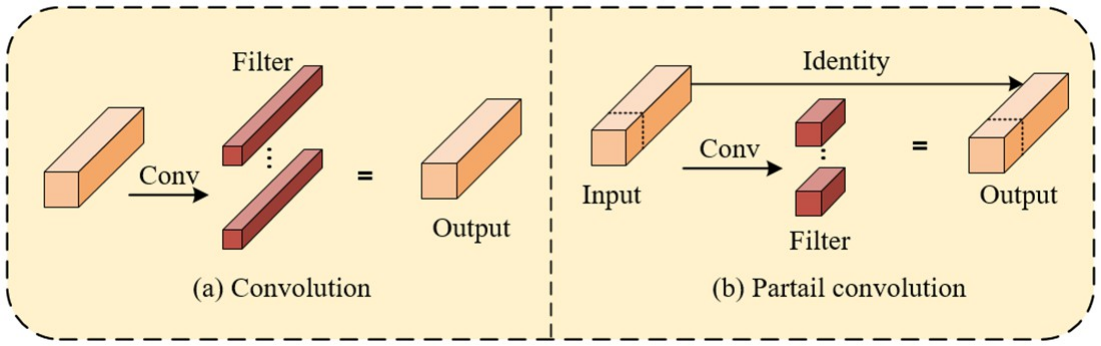
Comparison between normal convolution and partial convolution.

To address this issue, the paper introduces Partial Convolution (PConv) to replace some of the regular convolutions in the backbone of the YOLOv7-Tiny model, optimizing the network to enhance the efficiency and feature extraction capability of the hazardous chemical vehicle detection model. As shown in Fig 7, the spatial feature extraction difference between PConv and regular Conv lies in the fact that partial convolution operates only on a subset of contiguous channels to extract features and concatenate them with the remaining channels. This approach maintains the same number of channels in input and output feature maps while reducing computational and memory access costs [29]. Through this method, partial convolution can more effectively utilize information in the feature maps, thereby improving the detection efficiency and accuracy of the hazardous chemical vehicle detection model.

The algorithmic implementation of partial convolution is outlined in Table 1. In steps 1 and 2, the dimensions after segmentation and the dimensions before segmentation are accurately determined, providing the model with more flexible feature learning capabilities. In step 4, by invoking the split function, the input feature map *x* is segmented along dimension 1, resulting in outputs *x*1 and *x*2, representing the portions requiring convolution operations and those not requiring convolution operations, respectively. Next, in step 5, a 3 × 3 convolution is applied to *x*1 to extract features. Finally, in step 6, the concatenated function is called to concatenate the convolved *x*1 with *x*2, yielding the complete output feature.

**Table 1:** Algorithmic process of partial convolution.

Algorithm 1: Partial Conv
Input: Input feature map *x*; Number of input channels *C*_*in*_; Number of output channels *C*_*out*_.
Output: Output feature map *out*.
1: Calculate the portion for the convolution operation: *dim* − *conv* = *dim*/*n*; // n is set to 4 in this experiment, *dim* is 1;
2: Calculate the portion that does not require convolution operation: *dim* − *untouched* = *dim*—*dim* − *conv*;
3: Call the split Concat method: Function *forward* − *split* − *Concat*(*x*);
4: Split the input *x* along dimension 1 to obtain *x*1 and *x*2: *x*1, *x*2 = split(*x*, [*dim* − *conv*, *dim* − *untouched*], *dim* = 1);
5: Apply a 3 × 3 convolution to the segmented part *x*1: *x*1 = *partial* − *conv*3(*x*1);
6: Concatenate the convolved part *x*1 with the untouched part *x*2 along dimension 1 to obtain the final output *out*;
7: *out* = Concat((*x*1, *x*2), 1);
8: return *out*

Through the above process, we have successfully implemented the algorithm of partial convolution. This algorithm flexibly utilizes segmentation, convolution, and concatenation steps, effectively preserving the integrity of input feature map information while extracting crucial features. It has significantly improved the detection efficiency and accuracy of the hazardous chemical vehicle detection model. This innovative approach holds great significance in addressing challenges in scenarios with large-scale and dense traffic or real-time environments, providing an effective solution for the hazardous chemical vehicle detection field.

### Loss functions

The YOLOv7-Tiny algorithm utilizes the CIoU (Complete Intersection over Union) bounding box regression loss function, including penalties for aspect ratios. However, when the predicted bounding box’s aspect ratio matches that of the ground truth bounding box, the CIoU loss function becomes ineffective, resulting in a lack of stability in the model. In hazardous chemical vehicle detection tasks, training data inevitably contains low-quality samples due to geometric factors such as distance and aspect ratios, which can degrade the model’s generalization capability. Bounding Box Regression (BBR) plays a crucial role in hazardous chemical vehicle detection tasks, and the definition of its loss function is essential for improving model performance. Traditional BBR methods typically focus on enhancing fitting capabilities on high-quality samples. However, an excessive emphasis on high-quality samples may lead to neglecting the impact on low-quality samples, thereby reducing the accuracy of object localization on low-quality samples.

To address this issue, this study introduces an improved Weighted IoU (WIoU) bounding box loss function with a dynamically non-monotonic focusing mechanism. This enhanced feature ensures that the loss function remains stable and effective even when the aspect ratio of the predicted bounding box matches that of the ground truth bounding box. The dynamically non-monotonic focusing mechanism enables the model to better adapt to various scenes and object shapes, thereby improving the accuracy and stability of the YOLOv7-Tiny algorithm model in object detection.

Specifically, this paper adopts WIoU-v1, aiming to balance the training effects of different quality samples through a dynamic modulation mechanism. By reducing the training intervention on high-quality samples and lowering the penalty for geometric factors, the model relies more on the anchor box itself rather than contextual features for prediction. This strategy enhances the robustness and generalization performance of the model, enabling it to better adapt to hazardous material vehicle targets of different shapes and positions, ultimately improving the detection accuracy. The definition of the WIoU loss function is as follows:

$$\begin{array}{c} L_{WIoUv1}=R_{WIoU}L_{IoU} \end{array}$$ (9)

$$\begin{array}{c} L_{IoU}=1-\frac{Bbox\cap Tbox}{Bbox\cup Tbox} \end{array}$$ (10)

$$\begin{array}{c} R_{WIoU}=exp\left(\frac{{\left(x-x_{gt}\right)}^{2}+{\left(y-y_{gt}\right)}^{2}}{{\left(W_{g}^{2}+H_{g}^{2}\right)}^{*}}\right) \end{array}$$ (11)

**Fig 8:**
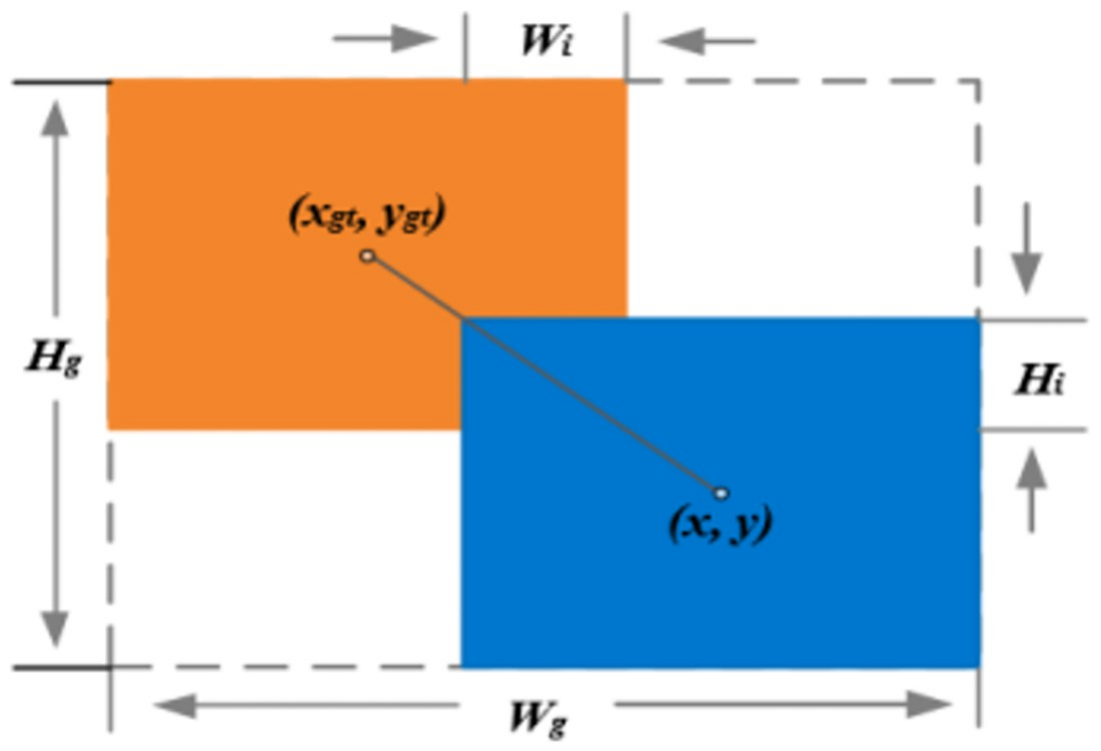
Intersection and juxtaposition of anchor frame and target frame.

Whereas from Eq (11), it can be observed that the range of *R*_*WIoU*_ is [1, e), aiming to significantly enhance the *L*_*IoU*_ of ordinary quality anchor boxes. Meanwhile, in Eq (10), *L*_*IoU*_ (Loss Intersection over Union) takes values in the range [0, 1]. Relative to *R*_*WIoU*_, the design of *L*_*IoU*_ aims to significantly decrease the *R*_*WIoU*_ of high-quality anchor boxes, especially when there is a high overlap rate between the predicted box and the ground truth box, and the distance between their center coordinates is small [30]. When there is an overlap between *Bbox* (predicted box) and *Tbox* (ground truth box), *L*_*IoU*_ focuses on the distance between their center points. This adjustment of the loss allows it to prioritize the accurate location of the target, not solely relying on the overlap. This helps the model better adapt to accurately predict the target boundary. Here, *x* and *y* represent the coordinates of the center point of the predicted box, *x*_*gt*_ and *y*_*gt*_ are the coordinates of the center point of the ground truth box, *W*_*g*_ and *H*_*g*_ represent the dimensions of the minimum bounding box. To avoid gradient hindrance in convergence, the superscript * indicates the separation of *W*_*g*_ and *H*_*g*_ from the computation graph, effectively eliminating factors hindering convergence. For detailed parameter meanings, refer to Fig 8.

As the WIoU loss function is dynamic, allowing for the dynamic adjustment of gradient gain allocation strategy based on the current situation at each moment, it effectively addresses the issue of sample quality imbalance preventing accurate matching of regression boxes. Therefore, this paper adopts the WIoU loss function to replace the original CIoU in the network, resolving the problem caused by the imbalance in sample quality.

## Experiments and discussion

### Experimental environment and parameter settings

For the experiments in this paper, we conducted on a Windows 10-based computer equipped with an NVIDIA Tesla V100 SXM2 graphics card boasting 16 GB of memory. The PyTorch deep learning framework, version 1.8.0, was selected as the development environment, and CUDA 10.2.89 was utilized to accelerate the training process. All models were trained from scratch without the use of any pre-trained models. Additionally, Python interpreter version 3.8 along with the SGD optimizer were employed to adjust the model parameters. Table 2 presents specific settings of key experimental parameters.

**Table 2:** Experimental parameter configuration.

Batch size	Epoch	Ir0	Weight decay	Momentum	Input size
32	100	0.01	0.0005	0.937	640

### Data preprocessing

**Fig 9:**
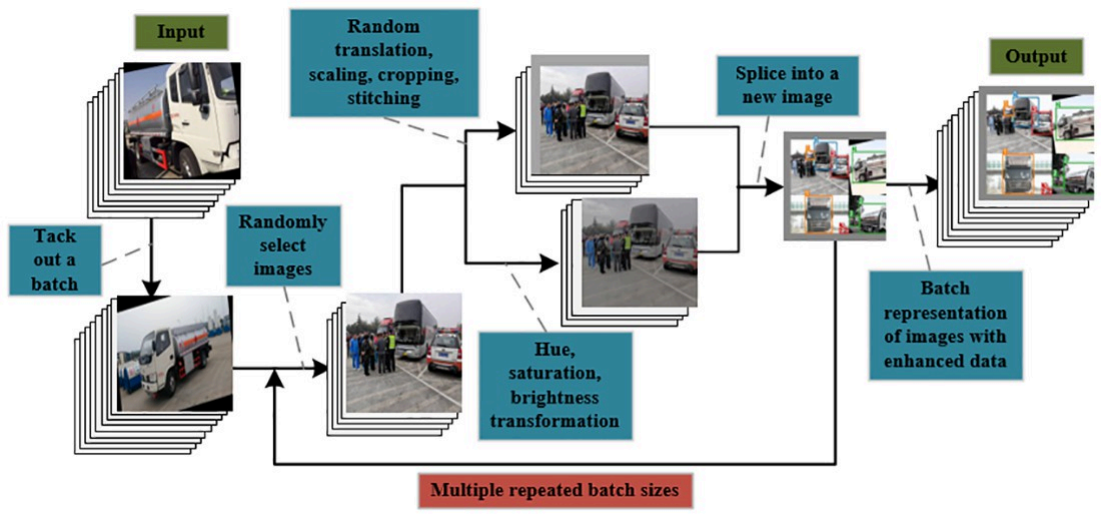
Flow chart of mosaic technology. Reprinted from [] under a CC BY license, with permission from [Pengcheng Zhu], original copyright [2023].

To improve the generalization ability and robustness of the model, we have used the mosaic technique to enhance the original data. The mosaic technique is an image data enhancement operation that transforms the image by dividing the original image into small blocks and recombining them. Each small piece is stitched together to form generate a new image sample after operations such as panning, scaling, cropping, stitching, and transforming hue, brightness, and saturation. This operation simulates visual changes in the real world, such as rotation, scaling, and translation of objects, enabling the model to obtain more information from the original image and improving the model’s ability to generalize to different scenes. Taking the hazardous chemical vehicle dataset used in this paper as an example, the mosaic technique is shown schematically in Fig 9.

By applying the mosaic technique, it is possible to increase the diversity of the dataset, and it helps the model to better cope with the task of target detection of hazardous chemical vehicles in various visual variations and complex scenarios.

### Experimental results and analysis

#### Analysis of training results

**Fig 10:**
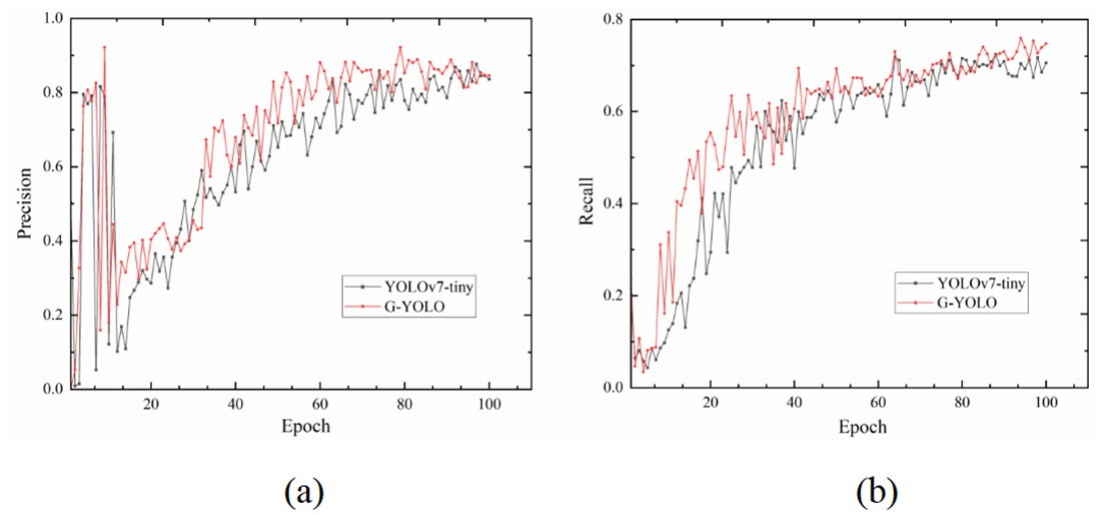
Training results of YOLOv7-tiny model and G-YOLO model on hazardous materials vehicle dataset. (a) Precision Curves Comparison; (b) Recall Curves Comparison.

**Fig 11:**
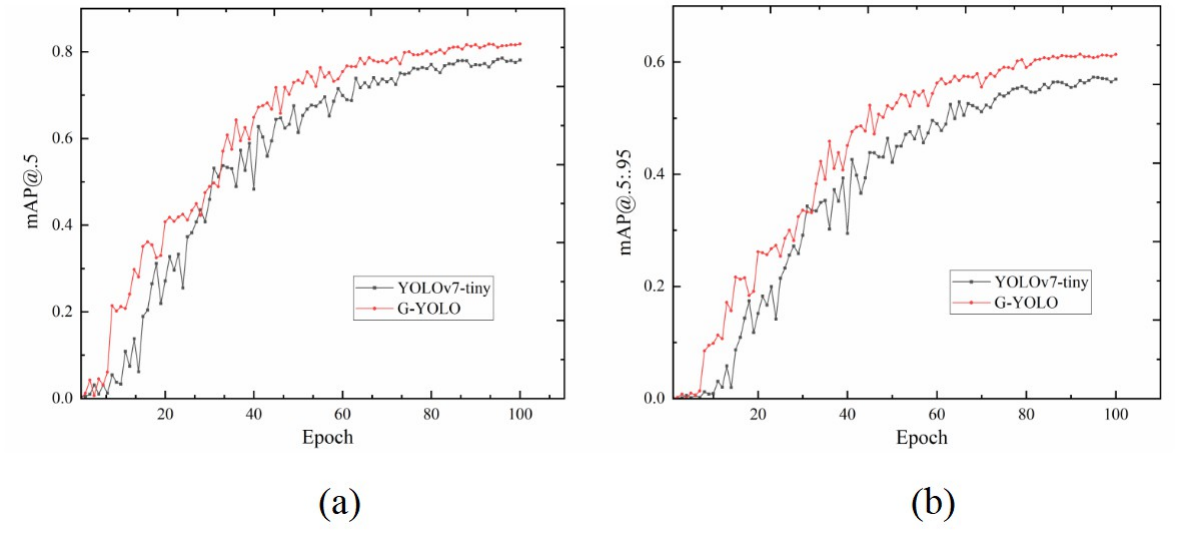
Training results of YOLOv7-tiny model and G-YOLO model on hazardous materials vehicle dataset. (a) mAP@.5 Curves Comparison; (b) mAP@.5: .95 Curves Comparison.

The YOLOv7-tiny model and the G-YOLO model were trained on the hazardous materials vehicle dataset, and the training results were initially evaluated using Precision, Recall, mAP@.5, and mAP@.5: .95 as metrics, and the comparison curves of the training results are shown in Figs 10 and 11.

As can be seen from Fig 10, the G-YOLO model outperforms the traditional YOLOv7-Tiny model on both the recall curve and the precision curve. From the recall curve, it can be seen that the G-YOLO model can identify more positive cases, i.e., more hazardous vehicles, under the same conditions. The observation of the precision curve shows that under the same conditions, the G-YOLO model can predict more true positive samples, i.e., a higher proportion of positive examples in the prediction results. This further proves the effectiveness of the G-YOLO model in detecting hazardous materials vehicles.

In order to further assess the validity of the G-YOLO model, mAP@.5 and mAP@.5: .95 of the model are analyzed in this paper, as shown in Fig 11.

The experimental results demonstrate the significant advantages of the G-YOLO model over the conventional YOLOv7-Tiny on two key evaluation metrics, namely mAP@.5 and mAP@.5: .95. First, through a detailed analysis of mAP@.5, we observe that G-YOLO achieves a higher level of detection accuracy on the Hazardous Chemical Vehicle dataset. This not only indicates that G-YOLO can recognize targets more accurately, but also achieves a superior performance under a relatively relaxed IoU threshold. This is crucial to ensure the credibility and accuracy of target detection results, especially when dealing with difficult scenarios such as changing viewing angles, complex backgrounds, and occlusions. Meanwhile, by evaluating mAP@.5: .95, we note that G-YOLO also achieves significant performance improvements at higher IoU threshold ranges. This indicates that the G-YOLO model has a stronger generalization ability for high-precision target detection and can maintain efficient detection accuracy under more demanding matching conditions. This is of great practical significance for applications that require high detection accuracy in some real-world scenarios, especially in areas such as hazardous materials vehicle monitoring.

**Fig 12:**
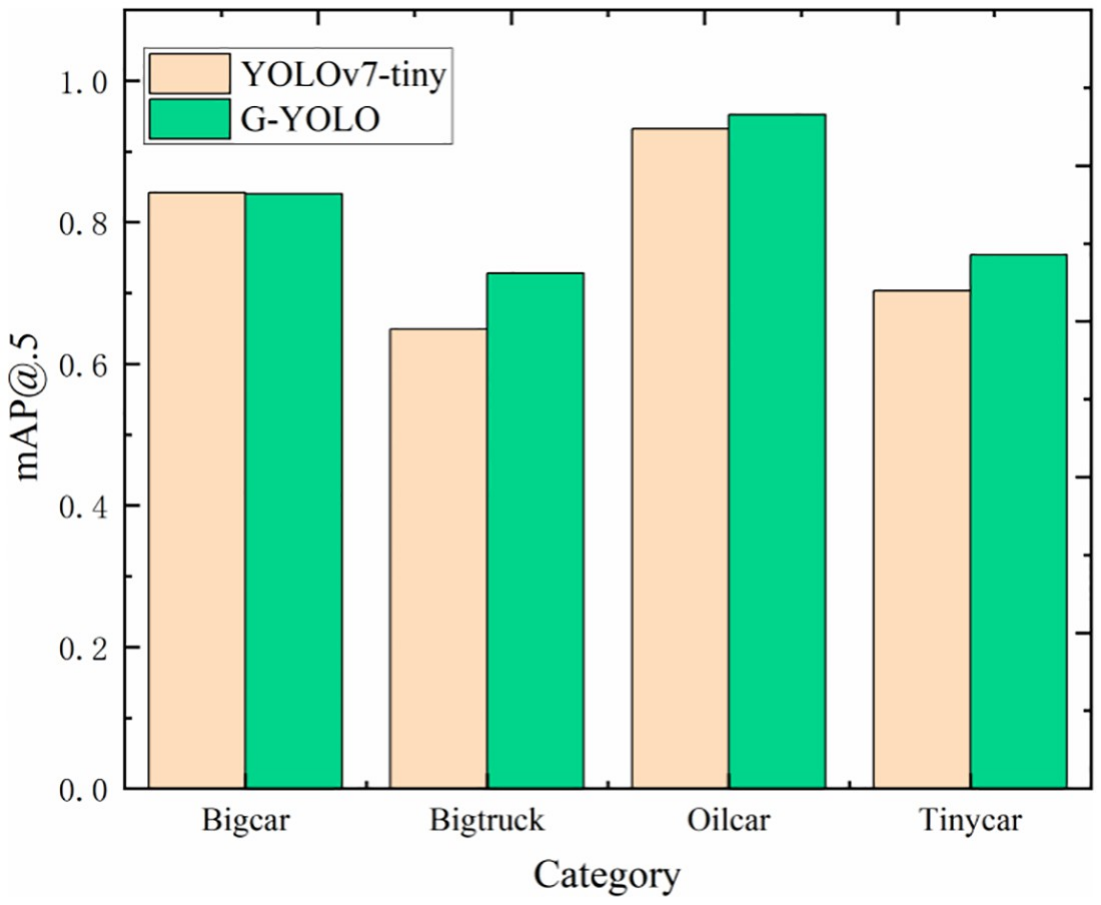
mAP@.5 comparison chart for various vehicle categories. Reprinted from [] under a CC BY license, with permission from [Pengcheng Zhu], original copyright [2023].

In addition, this study also conducted comparative experiments on four vehicles before and after the improvement of mAP@.5. The results of the experiments are shown in Fig 12.

Based on the mAP@.5 comparison plot in Fig 12, we observe that the G-YOLO model has significantly improved the detection performance of the G-YOLO model in each vehicle category relative to the YOLOv7-Tiny model on the Hazardous Chemical Vehicle dataset, with all three vehicle detection tasks except the Bigcar category presenting higher mAP@.5. This indicates that the G-YOLO model is more accurate and robust in the hazardous materials vehicle detection task. Thus, G-YOLO not only outperforms the benchmark model in terms of overall performance but also achieves higher accuracy in specific categories of detection.

#### Reasonableness validation of WIoU loss function selection

In this paper, to justify the selection of WIoU as the loss function, we conducted multiple comparison experiments between WIoU and other typical loss functions, such as SIoU, CIoU, EIoU, and GIoU, using the dataset presented in this study. The experimental results are summarized in Table 3.

**Table 3:** Comparison of multiple target detection experiments.

Loss Functions	mAP@.5	mAP@.5:.95
*CIoU*	78.0	56.3
*EIoU*	78.0	57.3
*SIoU*	77.1	57.3
*GIoU*	76.5	56.3
*WIoU*	**78.3**	**58.0**

The WIoU loss function, in contrast to several other loss functions, demonstrates higher mAP@.5 and mAP@.5: .95 values. This indicates that the model is more inclined to learn the association between the anchor frame and the target, relying more confidently on the anchor frame information for target detection. Consequently, the model exhibits improved robustness in the presence of various geometric variations and possesses a better generalization ability to adapt to hazardous material (hazmat) vehicle targets of different shapes and locations.

#### Ablation experiments

To validate the effectiveness of the proposed improvement method, we conducted a series of ablation experiments for comparative analysis, as shown in Table 4. The experiments were performed on the same dataset used in this paper, and to ensure accuracy and comparability, we used identical parameter settings. The table below presents the performance of the improved model and the YOLOv7-tiny model across various metrics.

**Table 4:** Comparison of ablation experiment results of models on hazardous chemical vehicle dataset.

Models	WIoU	PConv	E-GhostV2	Precision	Recall	mAP@.5	mAP@.5:.95	Para/M	FPS
*YOLOv*7 − *tiny*				85.2	69.2	78.2	57.3	6.03	263.16
*A*	✓			84.6	70.9	78.8	58.3	6.03	294.12
*B*		✓		88.2	67.8	78.5	58.6	6.11	303.03
*C*			✓	90.2	66.3	79.2	60.7	5.69	250.00
*G* − *YOLO*	✓	✓	✓	**83.8**	**74.4**	**81.5**	**61.3**	**5.78**	**256.42**

As can be seen from Table 4, the G-YOLO algorithm achieves significant improvements in all evaluation metrics. group A experiments show that replacing the CIoU loss function of the original model with the WIoU loss function leads to a 1.75% improvement in mAP@.5: .95 along with an 11.76% improvement in FPS, indicating that the introduction of the WIoU loss function effectively solves the problem of solving the target localization problem that is caused by blindly emphasizing high-quality samples. Group B experiments use partial convolution to replace part of the regular convolution of the YOLOv7-Tiny network backbone, which increases the number of parameters in the model, but the mAP@.5: .95 and precision of the model increase by 2.27% and 3.52%, respectively, and the FPS of the model improves by 15.15%, which indicates that the partial convolution not only improves the feature extraction capability of the model effectively but also improves the FPS of the model. improves the feature extraction ability of the model, but also successfully reduces the computational redundancy and the number of memory accesses, which significantly improves the detection efficiency of the model. The experiments in group C use the E-GhostV2 network introduced to the original model’s trunk and neck, which not only reduces the number of the model’s parameters by 5.64% but also improves the model’s detection mAP and precision to a certain extent. This demonstrates that the E-GhostV2 network can better capture the dependencies between pixels of long-range spatial locations of hazardous materials vehicles and improve the detection performance of the model while maintaining the light weight.

The last set of experiments shows the training results of the G-YOLO algorithm model on the dataset, and the G-YOLO model performs well in multiple sets of experiments, reaching a mAP@.5 of 81.5%, which is an improvement of 4.22% compared to the previous one. The average accuracy at mAP@.5: .95 achieved an improvement of 6.98%. These results indicate that the improved algorithm achieves better performance on the target detection task. At the same time, the number of parameters of the improved model decreased by 4.15%. This means that the model becomes more lightweight with lower storage and computation costs while maintaining higher performance. This is important for deploying and applying the model in resource-constrained environments.

To verify the effectiveness of the improved module proposed in this paper and the generalization ability of the improved model, we conducted ablation experiments on the Cars Detection dataset and the Traffic Detection dataset, and the experimental results are shown in Table 5.

**Table 5:** The effects of different modules in YOLOv7-Tiny for the cars detection dataset and traffic detection dataset.

Datasets	Models	WIoU	PConv	E-GhostV2	Precision	Recall	mAP@.5	mAP@.5:.95
*CarsDetection*	YOLOv7-tiny				29.5	41.2	27.4	12.3
	A	✓			35.0	35.8	27.0	14.2
	B		✓		30.5	34.3	28.5	13.9
	C			✓	36.0	38.6	34.4	19.1
	G-YOLO	✓	✓	✓	**42.2**	**51.6**	**41.6**	**23.7**
*TrafficDetection*	YOLOv7-tiny				51.7	36.9	38.0	23.9
	A	✓			55.0	37.1	39.1	24.9
	B		✓		51.0	38.4	39.4	25.0
	C			✓	47.5	36.8	36.2	21.0
	G-YOLO	✓	✓	✓	**56.7**	**34.2**	**39.0**	**22.5**

From Table 5, it can be seen that the G-YOLO model on the Cars Detection dataset has some degree of improvement in precision, recall, and mAP, which are 43.1%, 25.2%, 51.8%, and 92.7% respectively. Meanwhile, the model improves by 9.7% and 2.6% in precision and mAP@.5 on the Traffic Detection dataset, respectively.

Not only that, the introduction of different modules also enhances the detection results to different degrees. Specifically, in Group A experiments, the introduction of the WIoU loss function improves the precision of the YOLOv-Tiny model by 18.6% and 6.3% on the two datasets, which further verifies the ability of WIoU to effectively solve the problem that the regression frames cannot be accurately matched due to the imbalance in sample quality. In Group B experiments, the introduction of partial convolution improves the preciseness of the original model on the two datasets. The introduction of partial convolution improves the mAP@.5 of the original model on the two datasets by 4.0% and 3.7%, respectively, while the mAP@.5: .95 also achieves an improvement of 13.0% and 4.6%, respectively, verifying that the introduction of this module can greatly improve the model’s ability of feature extraction. In Group C experiments, the introduction of E-GhostV2 does not improve the mAP@.5 of the Traffic Detection dataset, but the performance on the Cars Detection dataset is good, which improves the model’s precision, mAP@.5 and mAP@.5: .95 by 22.0%, 25.5% and 55.3%, respectively, and reduces the number of parameters by 5.5%. This further validates that the introduction of E-GhostV2 gives the model the effect of improving the model’s detection accuracy despite the decrease in the number of parameters.

In summary, the comparison experiments between the YOLOv7-Tiny model and the G-YOLO model on three different datasets, respectively, not only verified the effectiveness of the addition of each module in improving the model detection effect but also verified that the improved model has a good generalization ability. This emphasizes the robustness of the improved model in different scenarios and lays the foundation for its generalization in practical applications.

#### Comparison of visualization results

To visually demonstrate the detection performance of the G-YOLO algorithm model, validation was conducted on the validation sets of the Hazardous Chemical Vehicles dataset, the Cars Detection dataset, and the Traffic Detection dataset, comparing the original YOLOv7-Tiny model with the G-YOLO model. The validation examples are presented below.

**Fig 13:**
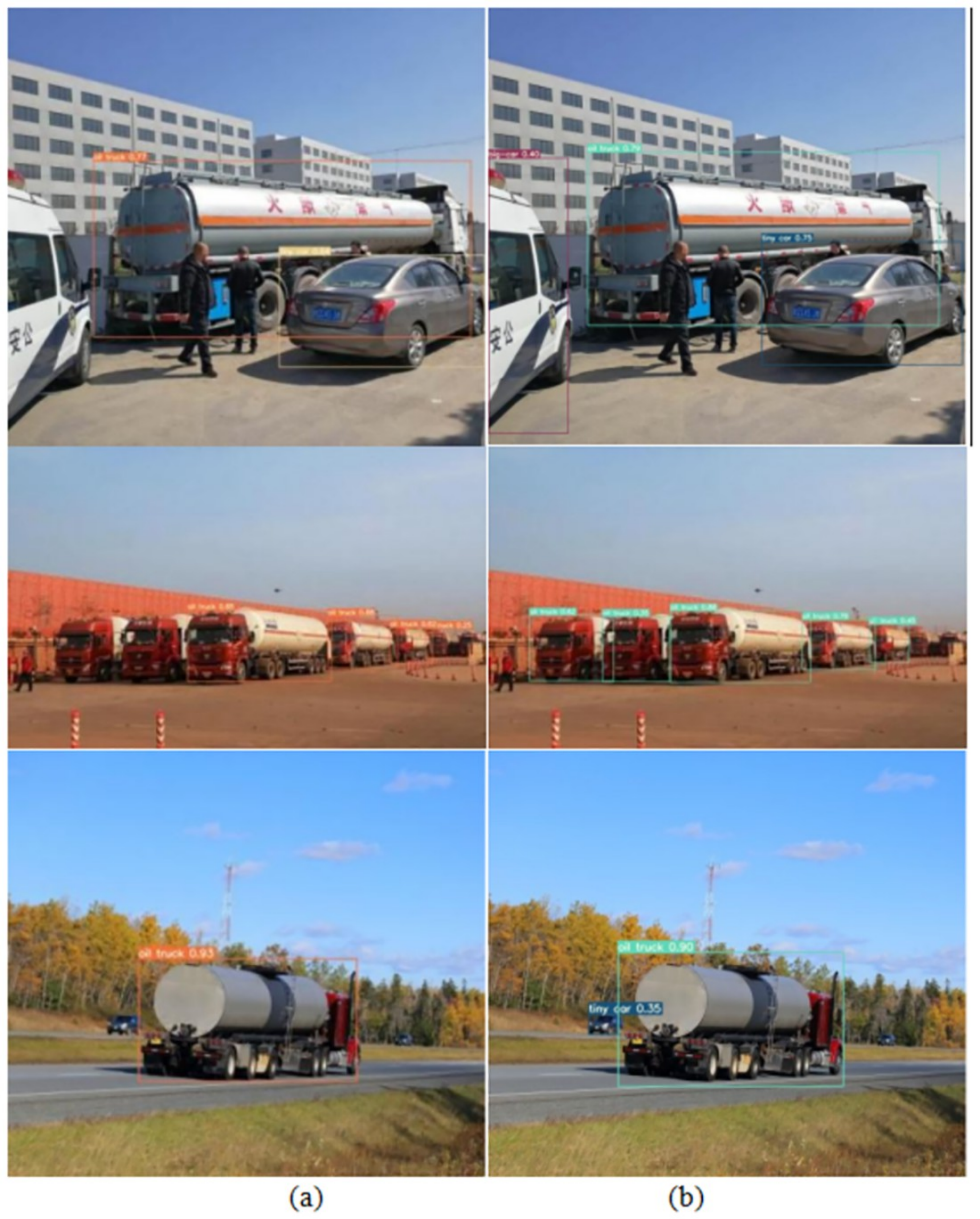
Validation instances of YOLOv7-tiny model and G-YOLO model on hazardous materials dataset. (a) shows the validation instances of the YOLOv7-tiny model; (b) shows the validation instances of the G-YOLO model. Reprinted from [] under a CC BY license, with permission from [Pengcheng Zhu], original copyright [2023].

By observing Fig 13, the differences between the G-YOLO model and the YOLOv7-Tiny model can be intuitively compared. We selected the Hazardous Chemical Vehicles dataset in real-world scenarios and demonstrated the detection results in scenarios with missed detections and dense scenes. Specifically, through detailed performance metric analysis (as shown in Table 4), the G-YOLO model exhibits higher accuracy and lower miss detection rate. As shown in Fig 13, in dense scenes, the G-YOLO model can accurately label vehicle information, effectively avoiding potential missed detection scenarios that may occur with the original model.

**Fig 14:**
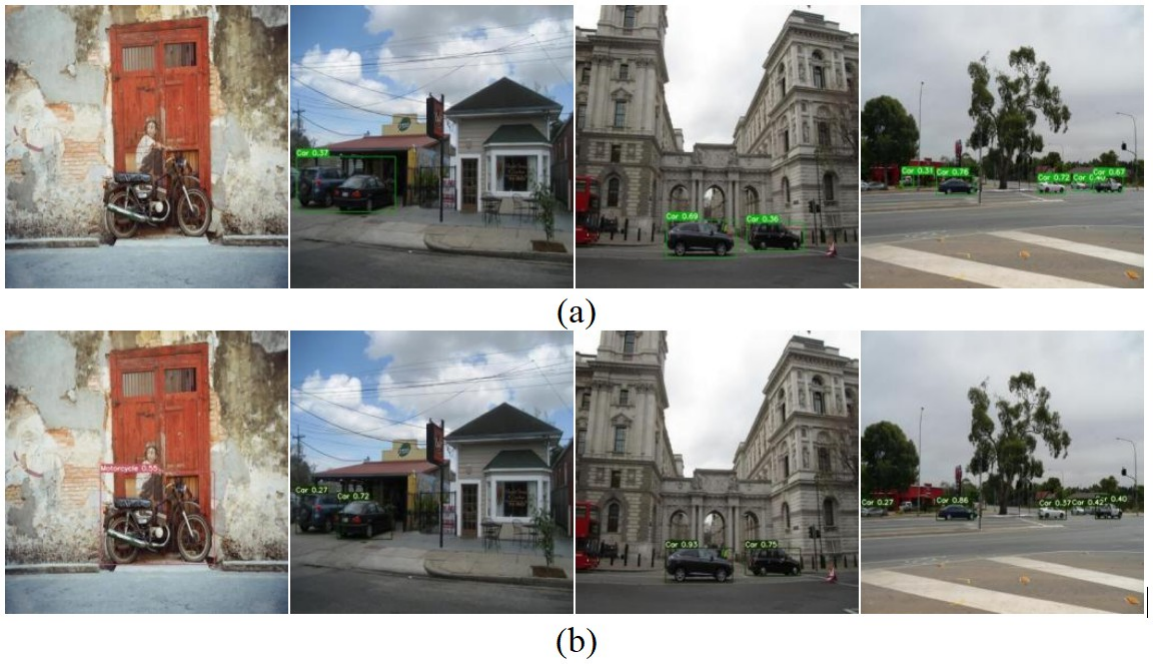
Validation instances of YOLOv7-tiny model and G-YOLO model on traffic detection dataset. (a) illustrates the validation instances of the YOLOv7-tiny model; (b) illustrates the validation instances of the G-YOLO model.

**Fig 15:**
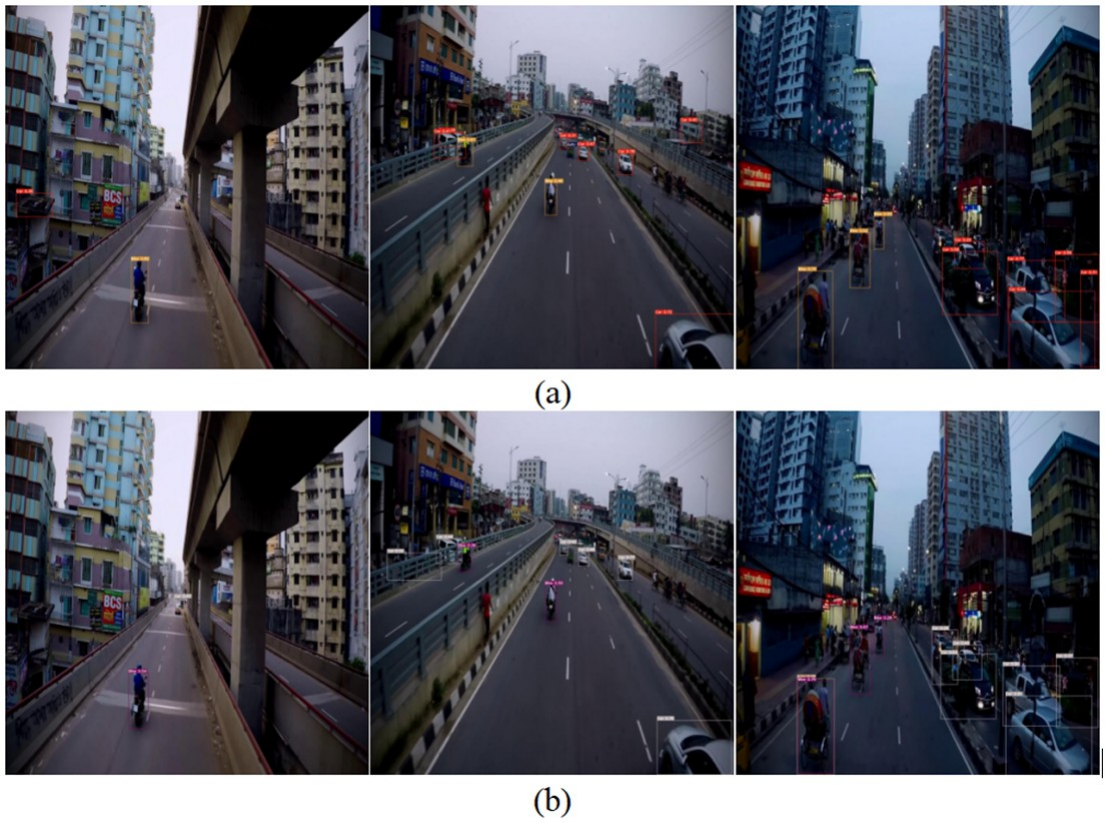
Validation instances of YOLOv7-tiny model and G-YOLO model on cars detection dataset. (a) illustrates the validation instances of the YOLOv7-tiny model; (b) illustrates the validation instances of the G-YOLO model.

Figs 14 and 15 depict validation examples of the YOLOv7-Tiny model and the G-YOLO algorithm model on the Cars Detection dataset and Traffic Detection dataset, respectively. Important insights can be gained through the observation of Fig 14. From the detection image on the far left, it is evident that G-YOLO successfully detected the Motorcycle target that was missed by the original model. Additionally, in other effective images, situations where the original model generated incorrect detections due to background interference can be observed. However, the enhanced network model successfully overcame these challenges, effectively identifying targets even in the presence of complex background interference. This further demonstrates the outstanding performance of the proposed improved model in handling complex backgrounds and target missed detections.

Fig 15, the images gradually decrease in brightness from left to right, leading to an increasing impact on the detection model. Meanwhile, there are numerous smaller targets in the images, further increasing the likelihood of missed detections for both the original and improved models. However, through a comparative observation of the images, it is found that the improved model exhibits a lower rate of missed detections compared to the original model and achieves a significant improvement in detection accuracy. This indicates that the proposed improved model has more robust performance in scenarios involving low brightness and small targets.

#### Comparison of multiple object detection experiments

To validate the outstanding performance of the G-YOLO model, this study comprehensively examined three lightweight models based on the YOLOv7-Tiny model, including MobileOne-YOLOv7-Tiny, MobileNetv3-YOLOv7-Tiny, and ShuffleNetV2-YOLOv7-Tiny [31–33]. Additionally, a comparison was made with two single-stage object detection models, namely SSD and YOLOX [34], and a two-stage object detection model, Faster R-CNN. Furthermore, the latest lightweight models, YOLOv8n, and its improved version Yolov8n-RepHGNetV2 [35], were included for comprehensive experimental comparison. The following sections will present the comparative results of multiple object detection experiments.Table 6 shows the comparison of experimental results of various target detection models.

**Table 6:** Comparison of multiple target detection experiments.

Models	mAP@.5	Recall	Para/M
*SSD*	64.5	59.8	26.29
*YOLOX*	71.8	65.8	54.21
*FasterR* − *CNN*	42.6	46.7	137.10
*MobileOne* − *YOLOv*7 − *tiny*	59.3	36.6	7.30
*ShuffleNetv*2 − *YOLOv*7 − *tiny*	69.0	63.7	4.31
*MobileNetv*3 − *YOLOv*7 − *tiny*	72.5	67.6	4.18
*YOLOv*8*n*	80.4	73.0	3.16
*Yolov*8*n* − *RepHGNetV*2	79.5	71.7	2.50
*G* − *YOLO*	**81.5**	**74.4**	**5.78**

The experimental results show a significant advantage in detection accuracy for the G-YOLO detection algorithm. In the experiments, the G-YOLO model achieved mAP@.5 and mAP@.5: 95 values of 81.5% and 61.3%, respectively. Compared to the YOLOv7-Tiny model, this represents an improvement of 4.22% and 6.98%, while reducing the number of parameters by 4.15%. This indicates that the G-YOLO model effectively enhances detection accuracy while reducing the model’s size, making it more suitable for edge applications. When compared to classical models such as SSD and Faster-RCNN, as well as several recent lightweight models (MobileNet-YOLOv7-Tiny, MobileNetv3-YOLOv7-Tiny, ShuffleNetV2-YOLOv7-Tiny, Yolov8n, and YOLOv8n-RepHGNetV2), the G-YOLO model outperforms them in the detection of dangerous chemical vehicles. It achieves high precision and real-time objectives, further validating the outstanding performance of the G-YOLO algorithm in the field of object detection.

## Conclusion

Dangerous chemical vehicles in road transportation pose extremely high risks. Real-time and accurate detection of these vehicles can effectively identify traffic accidents and prompt timely responses, thereby preventing casualties and unnecessary property losses. To address the challenges of low detection accuracy and weak real-time performance in traditional methods, this paper proposes a novel detection algorithm. Firstly, the lightweight E-GhostV2 network is integrated into the YOLOv7-Tiny architecture, optimizing its backbone and neck. This improvement aims to accurately capture distant spatial dependencies between pixels of hazardous substance vehicles, especially in complex backgrounds, significantly improving the model’s detection accuracy. Subsequently, a fast and efficient PConv is introduced into the model’s backbone. This strategy reduces computational redundancy, lowers memory access, and enhances the model’s operational efficiency and feature extraction capabilities. Additionally, to address the problem of decreased target localization performance due to excessive emphasis on high-quality samples, this paper adopts the WIoU loss function, further enhancing the model’s detection capabilities. Experimental results show that the G-YOLO model not only reduces the number of parameters but also correctly identifies and accurately locates dangerous chemical vehicles in complex scenes. This research takes a crucial step towards improving the accuracy of lightweight models in object recognition, potentially enhancing performance in low-power devices for precise vehicle detection tasks in complex scenarios. The findings of this study have practical implications for target detection in real-world scenarios with performance-constrained devices. Therefore, maintaining a balance between improving detection accuracy and model complexity is crucial.

Future work will focus on developing more efficient target detection model algorithms to further extend the depth of this research. Specifically, efforts will be concentrated on enhancing the model’s performance in handling challenging complex backgrounds to address the confusion caused by backgrounds. Additionally, optimization of the model’s detection effectiveness for smaller targets will be pursued to meet the demand for high-precision detection of small targets in practical scenarios. Future research directions also include further improving the real-time performance of the model to adapt to the requirements of various real-world application scenarios. This will contribute to better applying our research outcomes to real-world scenarios and driving the development of target detection technology across various domains.
